# Integrating fish swimming abilities into rapid road crossing barrier assessment: Case studies in the southeastern United States

**DOI:** 10.1371/journal.pone.0298911

**Published:** 2024-02-28

**Authors:** Ridge Sliger, Jessica Graham, Kathleen Hoenke, Matthew E. Kimball, Kenneth A. Sterling, Brandon K. Peoples

**Affiliations:** 1 Department of Forestry and Environmental Conservation, Clemson University, Clemson, SC, United States of America; 2 St. Andrew and St. Joseph Bays Estuary Program, Florida State University, Panama City, FL, United States of America; 3 Southeast Aquatic Resources Partnership, Washington, DC, United States of America; 4 Baruch Marine Field Laboratory, University of South Carolina, Georgetown, SC, United States of America; 5 USDA Forest Service, Okanogan-Wenatchee National Forest, Naches Ranger District, Naches, WA, United States of America; Penn State University, UNITED STATES

## Abstract

Many aquatic networks are fragmented by road crossing structures; remediating these barriers to allow fish passage is critical to restoring connectivity. Maximizing connectivity requires effective barrier identification and prioritization, but many barrier prioritization efforts do not consider swimming capabilities of target species. Given the many potential barriers within watersheds, inventory efforts integrating species-specific swimming speeds into rapid assessment protocols may allow for more accurate barrier removal prioritization. In this study, we demonstrate an approach for integrating fish swimming speeds into rapid barrier assessment and illustrate its utility via two case studies. We measured critical swimming speeds (*U*_*crit*_) of two stream-resident fish species with very different swimming modes: Yoknapatawpha Darter (*Etheostoma faulkneri*), an at-risk species whose current distribution is restricted to highly degraded habitat, and Bluehead Chub (*Nocomis leptocephalus*), an important host species for the federally endangered Carolina Heelsplitter mussel (*Lasmigona decorata*). We assessed potential barriers for Yoknapatawpha Darters in the Mississippi-Yocona River watershed, and Bluehead Chubs in the Stevens Creek watershed, South Carolina, USA. We integrated *U*_*crit*_ into the Southeast Aquatic Resources Partnership (SARP) barrier assessment protocol by estimating the proportion of individuals per species swimming at least as fast as the current through the assessed structures. Integrating *U*_*crit*_ estimates into the SARP protocol considerably increased barrier severity estimates and rankings only for Yoknapatawpha Darters in the Yocona River watershed. These results indicate the importance of including species-specific swimming abilities in rapid barrier assessments and the importance of species-watershed contexts in estimating where swimming speed information might be most important. Our method has broad application for those working to identify barriers more realistically to improve species-specific fish passage. This work represents a next step in improving rapid barrier assessments and could be improved by investigating how results change with different measurements of swimming abilities and structure characteristics.

## 1 Introduction

Aquatic networks across the world are highly fragmented by human-made barriers [1]. Road crossing structures such as culverts can prevent fish passage, causing fragmentation of streams and leading to population isolation and decline [2–4]. Fragmentation of streams is acute in the southeastern US due to pervasive human development of the landscape. Improving aquatic network connectivity will require significant efforts given the vast number of road crossing structures in the region. Reducing fragmentation will require identification and remediation of structures preventing passage. With limited budgets, organizations must prioritize barriers for mitigation to maximize management goals [5]. To improve prioritization efforts, various researchers have developed indices useful for identifying barriers and processes for prioritizing their removal across large spatial extents [6–9]. While useful, these indices can be improved by more detailed consideration of what structure types reduce fish passage, and how this reduction varies across species.

To mitigate the effects of barriers on fishes, we must first understand which road crossings are most likely to prevent their passage. Fish passage probability depends on the physical characteristics of the crossings and the swimming abilities of resident species [10–12]. Fishes differ widely in their life histories and swimming abilities, influencing the effects of habitat fragmentation caused by potential passage barriers [13, 14]. Integrating the swimming ability of focal species into barrier assessments can provide more accurate information concerning how severely certain structures affect fish passage. One method is to directly measure passage of tagged fish through structures [10, 15, 16]. Another approach involves using genetic methods to quantify population fragmentation in response to barriers [17–19]. These methods are useful for quantifying passage rates and population fragmentation, but they are more suited for estimating passage probability for small numbers of species in relation to small numbers of structures. The costs and labor required for these methods make them infeasible for assessing large numbers of barriers over large spatial extents, assessments which are valuable to watershed-level conservation.

One relatively simple method for estimating barrier severity is to quantify structure characteristics and compare them to fish swimming abilities [6, 20, 21]. However, this approach could be improved by integrating swimming ability data across a greater diversity of fishes to avoid two common assumptions: 1) that studied species can be used as surrogates for similar, unstudied species, and 2) swimming ability data collected using different methods can be used within the same framework. Making these assumptions allows researchers to take advantage of pre-existing fish performance data (e.g., [22]). However, while using such databases has been useful in bridging certain knowledge gaps, these assumptions may not always be met. Closely related species can have significantly different swimming speeds [13], and methodological choices in how swimming speed is measured can significantly alter swimming speed estimates [23]. Additionally, most fish swimming speed data is available for sportfishes or large bodied imperiled species [22, 24]; there is very little information on swimming speed of small bodied nongame species, which comprise the vast majority of fish diversity, particularly in the southeastern US [25].

We propose a rapid, standardizable approach for estimating swimming speeds and integrating them into barrier assessment scoring protocols. We demonstrate this approach using data from the Southeast Aquatic Resources Partnership (SARP), which maintains the largest repository for information on road-related barriers in the southeastern US [9]. This inventory includes crossings that were assessed with numerous protocols, though most were assessed using the standardized rapid SARP protocol. Numerous conservation groups use the results of these assessments, in conjunction with an associated web-based prioritization tool, to identify barriers to fish passage and prioritize their removal to maximize habitat connectivity for target species (e.g., The Nature Conservancy [7]). The SARP protocol provides the benefit of very rapid assessment, but it assumes road crossing structures affect passage probability for all species equally, regardless of species level variation in swimming ability. Integrating species-specific swimming speeds into the SARP protocol is a logical next step in improving the applicability of barrier assessments. We demonstrate our method using two case studies that quantify the relative severity of barriers within the ranges of two stream-resident fish species with very different swimming modes: Yoknapatawpha Darter (*Etheostoma faulkneri*) and Bluehead Chub (*Nocomis leptocephalus*; provisionally *N*. *interocularis* in our study area per [26]). Yoknapatawpha Darter is an at-risk species endemic to the highly altered Yocona River watershed of Mississippi, USA. Bluehead Chub is a common species in the southeastern US and is not imperiled. However, Bluehead Chub is an important host species for the critically endangered Carolina Heelsplitter mussel (*Lasmigona decorata*) in North and South Carolina, USA [27]. These case studies demonstrate applications for species that represent a diversity of conservation statuses and inhabit different geographies, highlighting the broad utility of our approach. Furthermore, integrating swimming speeds into the SARP protocol specifically can be particularly valuable, given its widespread use in cataloguing passage barriers well beyond the southeastern US.

## 2 Methods

**Fig 1:**
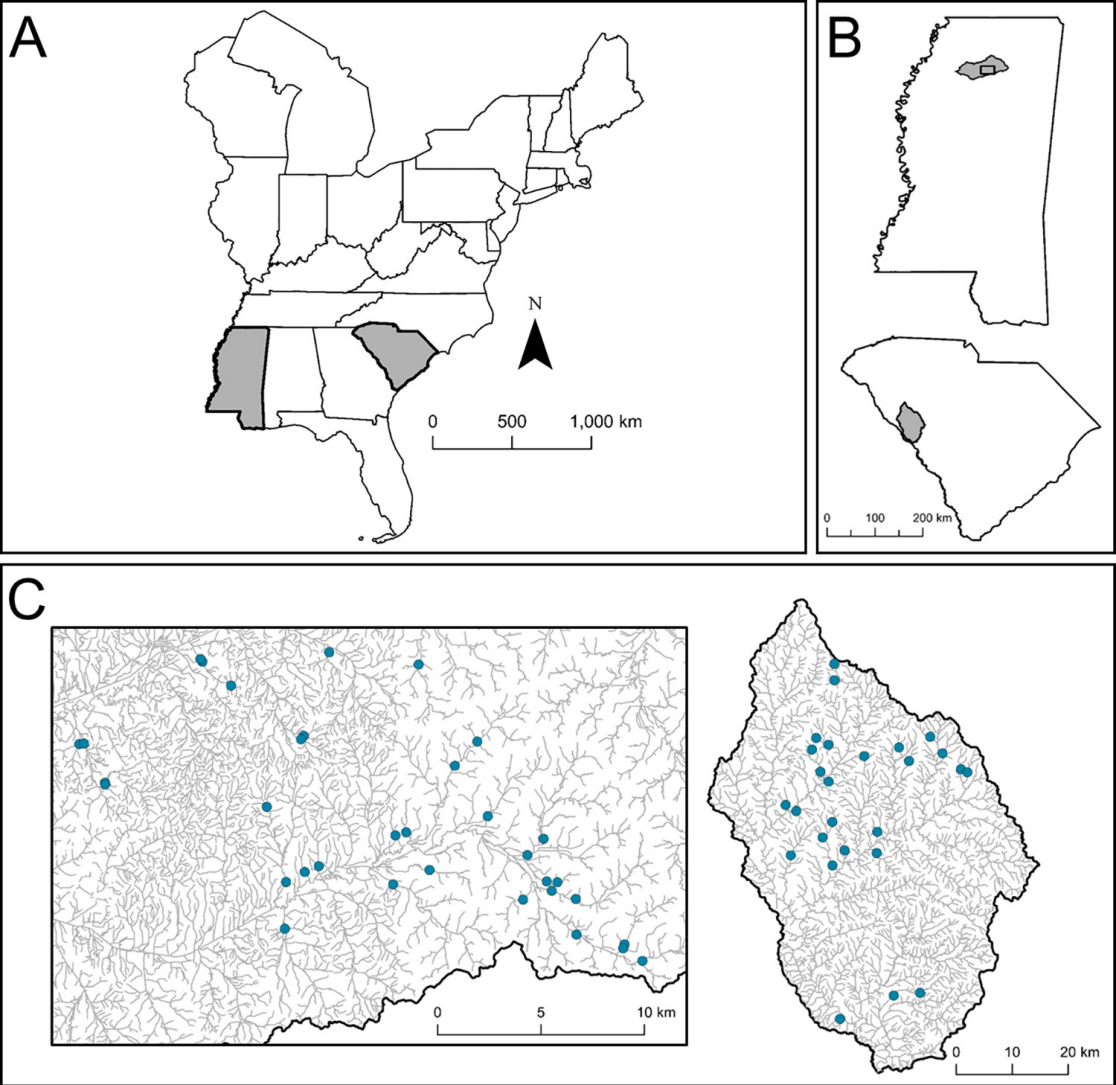
Road crossing locations. A) Mississippi (left) and South Carolina (right) shaded within the eastern US. B) The Yocona River (top) and Stevens Creek (bottom) watersheds shaded within their respective states. The black box in the Yocona River watershed outlines the area containing the assessed crossings. C) Locations of assessed crossing structures in the Yocona River (left; n = 34) and Stevens Creek (right; n = 26) watersheds are indicated by blue points.

Our two case studies focus on Yoknapatawpha Darters in the Yocona River watershed of Mississippi, and Bluehead Chubs in the Stevens Creek watershed of South Carolina (Fig 1A & 1B). In each watershed, we measured road crossing structures using the SARP assessment protocol, with additional measurements of current velocity. We estimated the prolonged swimming speeds of both species and compared them to structure flow velocities in their respective watersheds. Finally, we modified the SARP protocol with these comparisons and tested for differences between passage scores calculated by using the SARP standard protocol and our modified version.

### 2.1 Species and study areas

Yoknapatawpha Darter, recently split from the Yazoo Darter (*E*. *raneyi*), has a limited distribution in the Yocona River watershed of north-central Mississippi [28]. Having been largely channelized and incised, streams in this watershed are characterized by shallow, sandy, homogenous, unstable habitat, and are subject to flashy hydrology [29, 30]. Yazoo Darters in the Yocona River watershed had been considered in critical need of conservation [30, 31] before the Yoknapatawpha Darter was formally described [28]. The earliest account of Yoknapatawpha Darter habitat use indicates association with hard clay riffles in forested streams [32]. They are associated with instream cover in less-disturbed streams and anthropogenic structures in more disturbed streams [28]. Habitat degradation and fragmentation have reduced gene flow and population sizes [28, 33–36]. Given that swimming ability of this species is unknown, and that it has been negatively affected by habitat fragmentation, the Yoknapatawpha Darter is a particularly suitable model species for adding swimming ability to a rapid barrier assessment protocol.

For our second case study, we investigated potential barriers to Bluehead Chub movement in the Stevens Creek watershed, a tributary to the upper Savannah River, in the Sumter National Forest in South Carolina. Bluehead Chub occupies a broad distribution throughout the southeastern US and is often one of the most locally abundant fish species in streams where it occurs [37, 38]. Typical of the Piedmont ecoregion, the Stevens Creek watershed is characterized by a history of heavy deforestation and agricultural land use, which has caused significant gully formation [39, 40]. Forest cover is characterized by young hardwoods and pine with a mixed understory [39]. Although Bluehead Chub is not a species of greatest conservation need, it is a key host species for the federally endangered Carolina Heelsplitter, a freshwater mussel that attaches its glochidia to the gills of its hosts to develop into juveniles [27]. Carolina Heelsplitter is in severe need of conservation, and has been reduced to 11 highly disconnected populations in tributaries to the Pee-Dee, Santee, and Savannah Rivers of North and South Carolina [41]. Bluehead Chubs are frequently used as host fishes for Carolina Heelsplitter propagation efforts. Because juvenile dispersal of fish-parasitic mussels depends on host fish movement, structures that reduce passage for Bluehead Chub may so also for juvenile Carolina Heelsplitter. Therefore, understanding barriers to fish passage may also help us understand connectivity barriers to mussel species that require fish hosts. This inclusion demonstrates the utility of our methods to identifying barriers for taxa other than fish and allows for a contrast between a mid-column drift-feeding species and a more sedentary benthic species (i.e., Bluehead Chub and Yoknapatawpha Darter, respectively).

### 2.2 Fish swimming speed

We estimated prolonged swimming speeds by measuring critical swimming speed (*U*_*crit*_) of 18 Bluehead Chubs and 16 Yoknapatawpha Darters [42]. We placed individual fish into a closed chamber (28 L x 7.5 W x 7.5 H cm) of a Loligo Systems 5 L swim tunnel (www.loligosystems.com). After placement into the swim chamber, we acclimated individuals at no flow for 10 min, then at low flow (≤ 5 cm/s) for 5 min [13]. We calculated *U*_*crit*_ using the following equation derived from Brett [42]:

$$U_{crit}=U+(T/T_{i}*U_{i}),$$ (1)

where *U* is the penultimate velocity, *T* is the time swam in the final velocity, *T*_*i*_ is the velocity time interval (60 s), and *U*_*i*_ is the velocity increment (5 cm/s). We chose intervals of 60 s as we feel they represent the amount of time that a fish may use to traverse a crossing structure.

Swimming speed of Yoknapatawpha Darters was tested at the Private John Allen National Fish Hatchery in Tupelo, Mississippi. Darters were captured from an unnamed tributary in the Yocona River watershed, on February 4^th^ and March 26^th^, 2021. Hatchery personnel placed the darters in outdoor tanks with a temperature range of 20–24.4°C before placing them in indoor flow-through tanks with a temperature of approximately 18.6°C. During this holding period, fish were fed an *ad libitum* diet of bloodworms and then starved at least 12 hours before experimentation. After testing, they were rested in the tunnel at low flow for 5 minutes and then placed into a flow-through tank to ensure a return to normal behavior before being returned to their outdoor tank.

Bluehead Chubs were tested streamside at Six Mile Creek in the Clemson Experimental Forest in the Upper Savannah River Watershed. Specimens were collected with a backpack electrofisher, and then allowed to recover in a flow-through tub overnight before being used in swimming trials. Experimental water conditions during the swim trials were kept identical to those of the stream by pumping a constant supply of water from the stream into the swim tunnel by using a 0.33 horsepower sump pump. After experimentation, individuals were placed into an aerated bucket and allowed to recover for at least 20 min before being returned to the stream. All species were treated humanely in accordance with Clemson University Institutional Animal Care and Use Committee protocol number AUP2020-0068.

### 2.3 Road crossing assessments

We measured 34 crossings in the Yocona River watershed and 26 in the Stevens Creek watershed (Fig 1C) using protocols developed by the North Atlantic Aquatic Connectivity Collaborative (NAACC) and adapted for southeastern US streams by SARP [43]. The protocol measures characteristics considered to affect aquatic organism passage (Table 1). These measurements are designed to describe conditions around and through the structure in relation to a reference reach of the stream. Measurements include characterizations of the water depth, water velocity, and substrate inside the structure; conditions at and around the structure inlets and outlets; and the constriction, openness, and alignment of the structure in relation to the stream. These measurements are standardized, weighted, and summed to form a 0–1 index with lower values indicating more severe barriers to passage (S1 Appendix). This score does not estimate absolute passage probability but is meant to describe how severely a structure deviates from a natural stream. The overall score of the SARP standard protocol (SARP score) is calculated as the lesser value of either the outlet drop sub-score or the sum of the weighted sub-scores. For a crossing with multiple structures (e.g., a crossing consisting of three culverts), each structure receives a score as described above, and the overall road crossing score is the maximum value of each of the structure scores.

**Table 1:** Road crossing structure variables measured under the SARP assessment protocol. Continuous variables are identified, and categorical variables are given their associated levels. Each variable is given a possible score range (0–1 inclusive) and a weight. The SARP overall barrier score is composed of the minimum value between the weighted outlet drop sub-score, or the sum of all weighted sub-scores.

Variable	Level	Score	Weight
Outlet Drop	Continuous	0.00–1.00	0.161
Physical barriers	None	1.00	0.135
	Minor	0.80	
	Moderate	0.50	
	Severe	0.00	
Constriction	Severe	0.00	0.090
	Moderate	0.50	
	Spans only bankfull width	0.90	
	Spans full channel and banks	1.00	
Inlet grade	At stream grade	1.00	0.088
	Inlet drop	0.00	
	Perched	0.00	
	Clogged/collapsed/submerged	1.00	
	Unknown	1.00	
Water depth	Significantly deeper	0.50	0.082
	Significantly shallower	0.00	
	Comparable	1.00	
	Dry	1.00	
Water velocity	Significantly faster	0.00	0.080
	Significantly slower	0.50	
	Comparable	1.00	
	Dry	1.00	
Scour pool	Large	0.00	0.071
	Small	0.80	
	None	1.00	
Substrate matches stream	None	0.00	0.070
	Not appropriate	0.25	
	Contrasting	0.75	
	Comparable	1.00	
Substrate coverage	None	0.00	0.057
	25%	0.30	
	50%	0.50	
	75%	0.70	
	100%	1.00	
Openness	Continuous	0.00–1.00	0.052
Height	Continuous	0.00–1.00	0.045
Outlet armoring	Extensive	0.00	0.037
	Not extensive	0.50	
	None	1.00	
Internal structures	None	1.00	0.032
	Baffles/weirs	0.00	
	Supports	0.80	
	Other	1.00	

In addition to the SARP standard protocol, we quantified current velocity (cm/s) at each structure with a Hach FH950 portable flow meter to create a modified SARP score reflecting species-specific *U*_*crit*_. The singular current velocity associated with each structure was the greater value of either the inlet or outlet flow, each of which was selected as the lowest current velocity value of three evenly spaced measurements taken across the face of both the inlet and outlet at 60% depth when possible. We only measured water velocity at one depth to maintain protocol rapidity, and we chose this depth because it is standard across various assessments of streams. Additionally, many of the measured structures had little depth and no coarse substrate, and therefore, there would likely be little difference between mid-column and bottom current velocities when measured by commonly used probes. The overall score for this modified protocol (*U*_*crit*_ score) was calculated as the least value among the outlet drop sub-score, the sum of the weighted sub-scores, and the proportion of tested individuals having a *U*_*crit*_ at least as great as the current velocity through the structure. A full list of road crossing variables is given in Table 1, and example calculations are provided in A.1 of S1 Appendix.

### 2.4 Data analyses

After calculating SARP and *U*_*crit*_ scores for all road crossing structures, we used a paired t-test for each watershed to quantify any differences in barrier severity scores caused by integrating *U*_*crit*_ data into the SARP protocol.

## 3 Results

**Fig 2:**
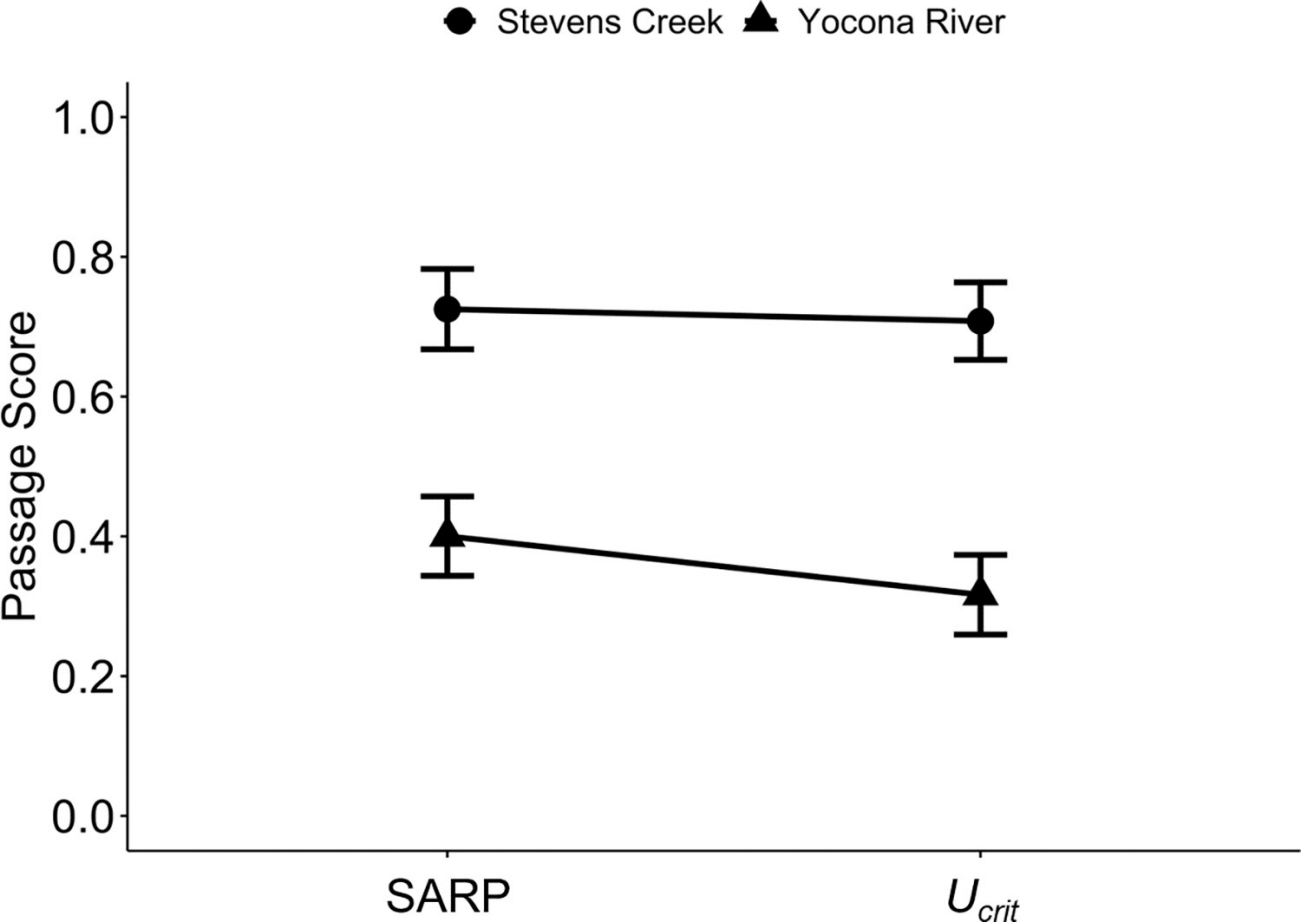
Road crossing passage scores (mean with one standard error) for the Stevens Creek and Yocona River watersheds before (SARP) and after (*U*_*crit*_) integrating critical swimming speed.

**Fig 3:**
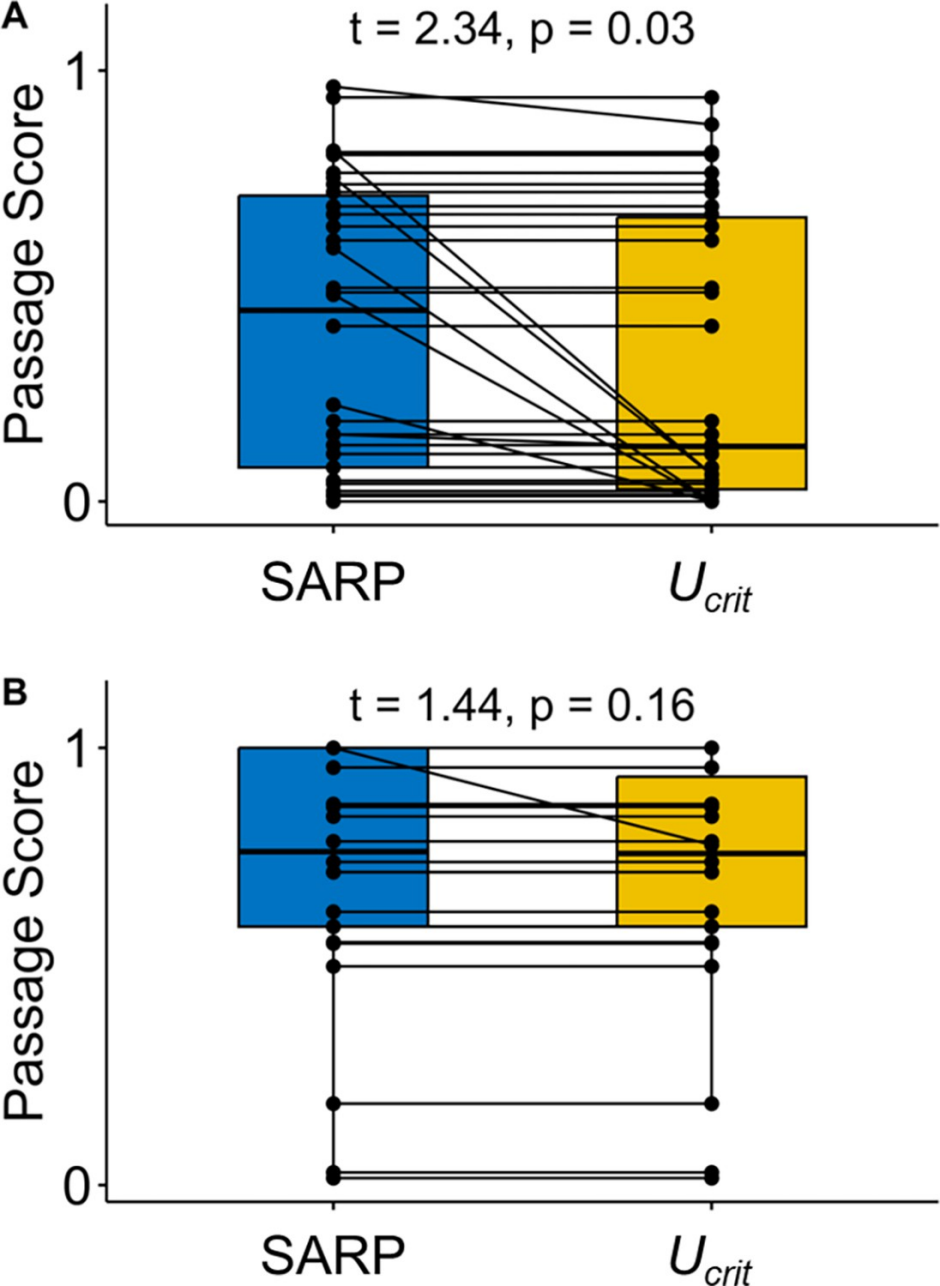
Individual road crossing passage scores in the Yocona River watershed (A) and Stevens Creek watershed (B) before (SARP) and after (*U*_*crit*_) integrating critical swimming speed for each crossing (unique crossings, points, are joined by black lines).

Expectedly, Yoknapatawpha Darters were slower swimmers than Bluehead Chubs (Table 2). In the Yocona River watershed, most of the characteristics measured by the SARP protocol had lower sub-scores than those measured in the Stevens Creek watershed (i.e., they were more severe; Table 3). Additionally, the structure current velocities were higher in the Yocona River watershed than in Stevens Creek. After considering the proportion of individuals with *U*_*crit*_ values higher than each structure current velocity in its watershed of residence, the overall barrier score (*U*_*crit*_ score) was significantly reduced from the SARP score in the Yocona River watershed, but not in the Stevens Creek watershed (S1 and S2 Tables, Figs 2 and 3). These results demonstrate how fish swimming and station-holding abilities, physical structure characteristics, and assessment protocols interact to create barrier severity estimates.

**Table 2:** Critical swimming speed results and testing conditions. Sample sizes, *U*_*crit*_ statistics, body size statistics, water temperature, and time of testing are given for each species.

Species	N	*U*_*crit*_ (cm/s)		Total Length (mm)		Water Temp (°C)	Testing Months
		mean	SE	mean	SE		
Yoknapatawpha Darter	16	46.9	4.0	55.3	1.7	18.6	June
Bluehead Chub	18	67.5	3.8	113.1	5.9	22.8	June and July

**Table 3:** Road crossing structure values for structures in the Yocona River (n = 34) and Stevens Creek (n = 26) watersheds. Variables include each of the 13 SARP sub-scores (italicized), current velocity (cm/s), the proportion of fish with *U*_*crit*_ at least as high as the current velocity (Proportion Passing), and the two overall scores (*).

Variable	Yocona River		Stevens Creek	
	mean	SE	mean	SE
*Outlet drop*	0.53	0.08	0.84	0.06
*Physical barriers*	0.81	0.06	0.82	0.07
*Constriction*	0.48	0.05	0.28	0.06
*Inlet grade*	0.94	0.04	0.92	0.06
*Water depth*	0.31	0.08	0.36	0.08
*Water velocity*	0.34	0.08	0.74	0.08
*Scour pool*	0.56	0.08	0.40	0.10
*Substrate matches stream*	0.21	0.07	0.39	0.10
*Substrate coverage*	0.14	0.05	0.35	0.09
*Openness*	0.97	0.01	0.96	0.02
*Height*	0.96	0.02	0.93	0.03
*Outlet armoring*	0.72	0.07	0.74	0.08
*Internal structures*	1.00	0.00	0.99	0.01
Current velocity	27.41	5.34	10.19	3.37
Proportion Passing	0.76	0.07	0.98	0.01
SARP Score*	0.40	0.06	0.73	0.06
Ucrit Score*	0.32	0.06	0.71	0.06

### 3.1 Fish swimming speeds

Yoknapatawpha Darter *U*_*crit*_ ranged from 16.8 to 79.4 cm/s with a mean of 46.9 ± 4.0 cm/s (± SE) (Table 2). Linear regression revealed no significant relationship between body size (total length, mm) and *U*_*crit*_ (*β* = 0.55 ± 0.60, *p* = 0.37, R^2^ = 0.06). The darter *U*_*crit*_ values reflect a combination of both swimming and station-holding behaviors. Typically, the darters began the trials by station-holding, angling their pectoral fins to provide downforce to maintain a benthic position and avoid being swept backwards. As velocities increased beyond their abilities to station-hold, they would swim in a bursting manner until reaching fatigue and becoming impinged on the swimming chamber screen. Whether station-holding or burst-swimming, darters remained on the bottom of the swimming chamber, without suspending themselves into the water column. After the water velocity was reduced to zero, the darters would rest at the back of the swimming chamber in the location at which they were impinged, until they were removed and placed into a bucket to recover.

Bluehead Chubs were relatively fast swimmers, with a *U*_*crit*_ ranging from 38.3 to 87.0 cm/s and a mean of 67.5 ± 3.8 (Table 2). Linear regression indicated that chub total length was positively related to *U*_*crit*_ (*β* = 0.36 ± 0.13, *p* = 0.017, R^2^ = 0.31). Chubs also typically began trials resting on the bottom of the swimming chamber. However, as water velocity was increased high enough to displace the fish, they swam against the flow and did not exhibit station-holding behavior. They were more active swimmers and remained suspended in the water column until reaching fatigue and becoming impinged on the swimming chamber screen. After the water velocity was reduced to zero, they would also rest on the bottom of the chamber until being placed in a bucket to recover.

### 3.2 Road crossing assessments

**Fig 4:**
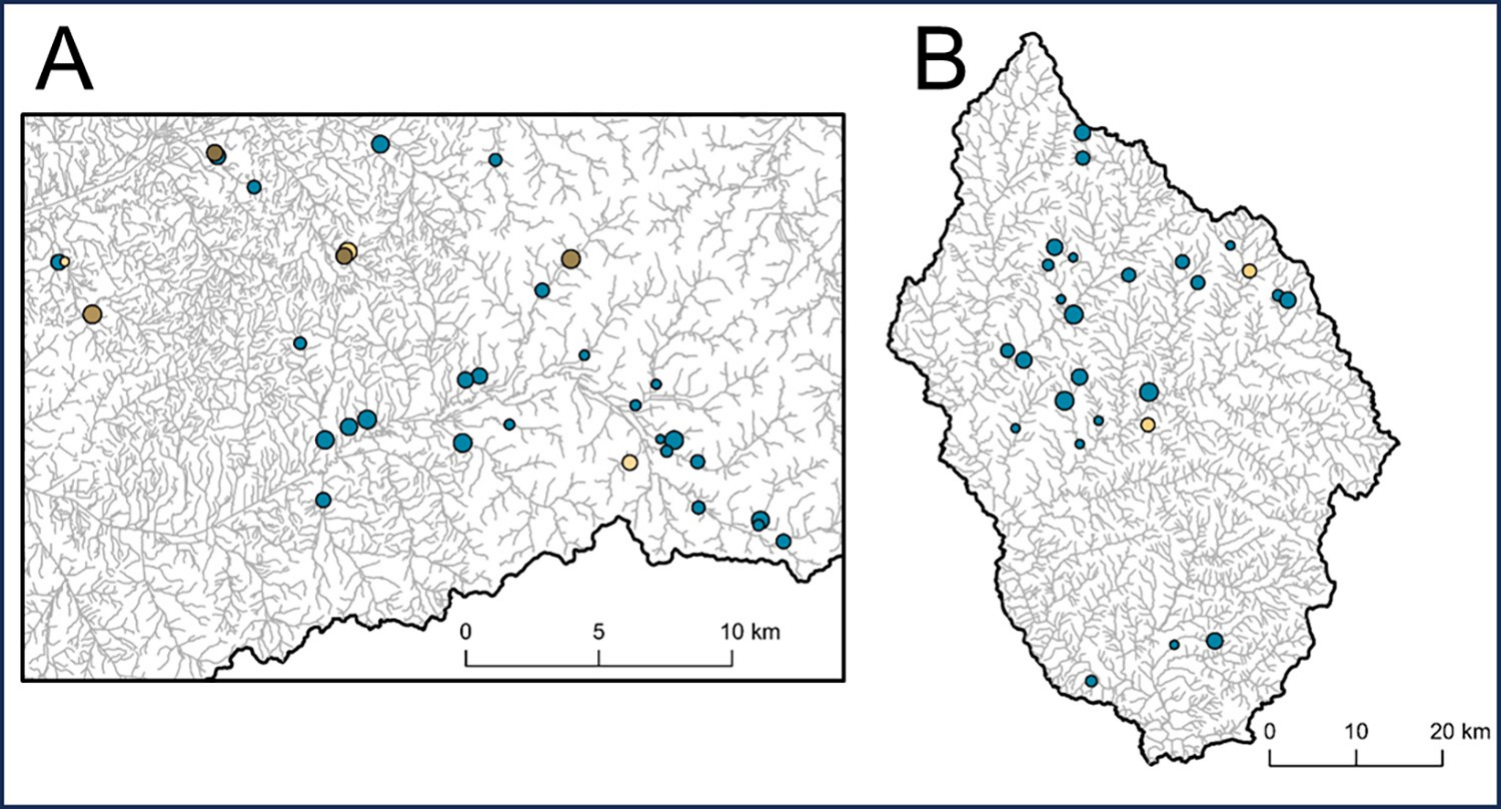
Assessed road crossing structures in the Yocona River (A) and Stevens Creek (B) watersheds after integrating critical swimming speed. Point size represents *U*_*crit*_ score, with larger points indicating more severe barriers. Blue points indicate crossings that did not change scores after *U*_*crit*_ integration. Yellow points indicate structures that had more severe scores after *U*_*crit*_ integration, with darker shades indicating greater differences from the initial SARP scores.

In the Yocona River watershed, the mean SARP score was 0.40 ± 0.06 (Table 3). Structures had particularly severe sub-scores for depth (0.31 ± 0.08), substrate matching (0.21 ± 0.07), substrate cover (0.14 ± 0.05), relative velocity (0.34 ± 0.08), and constriction (0.48 ± 0.05), indicating unfavorable conditions for fish passage. All other sub-scores had mean values greater than 0.5, demonstrating more favorable conditions for these variables. The mean structure current velocity was 27.4 ± 5.3 cm/s, and after accounting for the proportion of individuals swimming at least as fast as each structure’s current velocity, the mean *U*_*crit*_ score was reduced to 0.32 ± 0.06 (Figs 2–4). The paired t-test indicated that structure scores were significantly more severe, or greater barriers to passage, after integrating Yoknapatawpha Darter *U*_*crit*_ (*p* = 0.0252, S1 Table, Figs 2 and 3).

In the Stevens Creek watershed, the mean SARP score was 0.73 ± 0.06 (Table 3). The lowest sub-scores were those associated with depth (0.36 ± 0.08), scour (0.40 ± 0.10), substrate matching (0.39 ± 0.10), and substrate cover (0.35 ± 0.09). All other sub-scores had values greater than 0.7. The mean structure current velocity was a low 10.2 ± 3.4 cm/s, and after accounting for the proportion of individuals swimming at least as fast as each structure’s current velocity, the mean *U*_*crit*_ score was reduced to 0.71 ± 0.06 (Figs 2–4). However, the paired t-test indicated that structure scores were not significantly more severe after integrating Bluehead Chub *U*_*crit*_ (*p* = 0.1613, S1 Table, Figs 2 and 3).

## 4 Discussion

Integrating species-specific swimming speeds into the SARP rapid assessment protocol resulted in important changes to barrier severity estimates. From an applied standpoint, our most important finding is that integrating species-specific swimming speeds can dramatically alter not only barrier estimate values, but also barrier severity rankings. This result is particularly evident for Yoknapatawpha Darters in the Yocona River watershed. After including *U*_*crit*_ data, the estimated severity of these structures increased significantly. Additionally, the structures with the highest final estimated barrier severities (*U*_*crit*_ score) included structures across the spectrum of previously estimated barrier severity (SARP score). This strongly indicates that prioritization protocols that do not integrate target species life history (e.g., swimming ability, spawning phenology, or behavior) are likely to be misleading.

The results of integrating swimming speeds into crossing assessments were influenced by species-watershed contexts. The elements of swimming abilities and crossing structure characteristics interacted to yield different results for the example watersheds. Bluehead Chubs swam faster than Yoknapatawpha Darters, and before *U*_*crit*_ adjustments, barriers were identified as less severe in the Stevens Creek watershed. Because our case studies consisted of a slow-swimming species faced with high velocities and a fast-swimming species faced with low velocities, their results contrast to highlight the importance of including local context and swimming abilities of conservation targets in barrier assessment. This result demonstrates the importance of deciding which species-watershed combinations should be prioritized for barrier assessment. These decisions can be facilitated by knowledge of landscape-scale effects on road crossing characteristics. For example, in the Great Lakes Basin, estimated barrier severity increases with the gradient of the stream [6]. Likewise, estimated barrier severity increases with elevation in several watersheds in Florida [8]. In those contexts, researchers may choose to prioritize estimating the swimming ability of weaker swimmers in higher elevation watersheds.

Given the extent of habitat fragmentation caused by road crossing structures, various efforts have sought to improve barrier removal optimization [44–46]. However, before we prioritize candidate structures for removal, knowing their relative passability by key fish species will help with the ranking process. Directly measuring fish passage provides the most accurate barrier severity estimation, but is logistically difficult and primarily yields site-specific information (e.g., [10, 15, 47]). As such, these measurements are infeasible for very large barrier candidate sets such as the SARP inventory. Barrier assessment protocols such as the SARP protocol can be used to assess much larger candidate sets of barriers but may not yield accurate estimates of passability respective to target species. Other protocols fall on intermediate positions within the passage estimation accuracy vs. protocol useability tradeoff. For example, Coffman [20] developed a model that combines structure characteristics and coarse swimming abilities to infer passability. Januchowski-Hartley et al. [6] used water velocity and outlet drop data from 2,235 culverts and coarse swimming speed classifications to predict structure passability within the Great Lakes Basin. The FishXing software includes measurement protocols and literature-derived swimming abilities to estimate passability, and various studies use it as a standalone assessment or as a comparison against other methods [10, 21, 22, 44, 47]. To date, no single barrier assessment protocol has been identified as universally ideal, and barrier removal prioritization efforts will continue to benefit from protocols that combine specific swimming abilities, life history characteristics, and structure measurements over large spatial extents. Although some approaches have combined coarse structure measurements and fish swimming abilities, there is still a need to combine these types of information in novel ways for practical applications. Compared to some of the above approaches, the SARP protocol is intended to be a particularly rapid assessment. The approach we present here is a useful addition to existing frameworks in that they preserve the rapidity of the SARP protocol while allowing for simple integration of fish swimming speed estimates. We used point estimates of inlet and outlet current velocity measured only at mid-column. While more detailed measurements would have more thoroughly described the velocity profiles, they would have considerably increased assessment efforts at each structure. Such coarsely measured current velocity is a potential limitation of our results but is also a future opportunity for researching how current velocity measurements choices affect barrier estimates. Further research could be designed to determine how results may change when using different measurements of flow velocity, and how these changes differ for different species and watersheds.

It is important to emphasize that if we had used the best available swimming speed estimates from the literature, our results could have differed appreciably. Mean Yoknapatawpha Darter U_*crit*_ was 46. 9 cm/s, but the only swim speed estimates for *Etheostoma* species included in FishXing are 28.0, 29.6, and 31.2 cm/s for *E*. *whipplei*, *E*. *radiosum*, and *E*. *blennioides* respectively, none of which belong to the same clade as the Yoknapatawpha Darter (*Adonia*; [48]). No estimates are included for any *Nocomis* species [22]. Thus, our methods represent a way to maintain rapid structure assessments when new swimming speed estimates are required, potentially increasing biological realism for many barrier assessments while remaining relatively easy to use. Another benefit of our approach is that it allows for estimation of partial passability at each structure instead of only labeling them passable or impassable. However, our method could easily be adjusted to a more conservative approach in which barriers are considered impassable if the current velocity was greater than the mean swimming speed of the target species, for example. Depending on specific management goals or research objectives, swimming speed estimation could be applied in a myriad of ways. Indeed, there are many possible opportunities for applying our methods to species of different sizes, having greater size variation, or for which size may more strongly affect swimming ability. Regardless of these methodological choices, this increase in species-specific information can help to more efficiently prioritize resources used in species targeted barrier removal efforts.

One limitation of our approach is that barrier severity estimates are derived from current velocity measurements taken once during summer low flows. It does not incorporate temporal variability of the flow velocity in streams, which is known to affect passage in some contexts [49, 50]. However, inclusion of temporal variability in rapid assessments may not always be worth the additional effort, given that assessment periods do not always have notable effects on passage estimates, and rapid assessments would still serve as suitable screening processes for the worst barriers [49]. Additionally, flow variability can be more closely investigated when designing a replacement structure. Although rapid assessments can still be useful without consideration of flow variability, it should still be incorporated into passage barrier assessments in general. Structures in streams with different flow regimes will likely be negotiated by different species, given the importance of flow in structuring stream-resident fish assemblages [51–53]. Additionally, hydrologic variation can influence fish attraction to passable areas of potential barriers [54, 55]. For these reasons, the most informed barrier prioritizations should increase incorporation of flow variability. Our results are also limited in that they rely on swimming speed estimates taken from single populations at single times. However, physical characteristics varying with season and location can affect fish swimming ability. For example, water temperature varies widely with season and location and can affect fish swimming abilities significantly [56–58]. Future research would improve upon ours by measuring fish swimming speeds across multiple populations and seasons to match the contexts under which structures are being assessed.

Although there are various other species-specific abilities and behaviors that could, and eventually should, be integrated into barrier severity metrics, it is not currently feasible to identify what all characteristics are relevant to passage for all species in all watersheds, and to integrate that information into rapid assessments. Our methodology is a practical step towards increasing biological reality in a widely established, rapid barrier assessment protocol. Integrating even coarse swimming abilities into a rapid assessment such as the SARP protocol can better illuminate the contexts under which structures are likely to be passage barriers. Given the severity of habitat fragmentation caused by impassable road crossing structures, we encourage other researchers and managers to build upon our methodology depending on their specific connectivity goals. This could increase the efficiency of barrier removal prioritization efforts and establish more generalizability in species-watershed barrier patterns. Additionally, our methods are particularly useful for creating locally relevant barrier inventories, incorporating both structure characteristics and the abilities of the fish that negotiate them. These inventories are critical for the conservation of aquatic communities in areas with high levels of diversity and endemism, such as the southeastern US.

## Supporting information

S1 Appendix Road crossing scoring example. (DOCX)

S1 Table T-test results for paired comparisons of barrier scores before and after integrating Ucrit in each watershed. (DOCX)

S2 Table Overall scores for all measured road crossings. (DOCX)

S1 Data Crossing data. (CSV)

S2 Data Ucrit data. (CSV)

S3 Data Data definitions. (CSV)

## References

[1] Belletti B, Garcia de Leaniz C, Jones J, Bizzi S, Börger L, Segura G, et al. More than one million barriers fragment Europe’s rivers. Nature. 2020;588(7838): 436–441. doi: 10.1038/s41586-020-3005-2 33328667

[2] Fuller MR, Doyle MW, Strayer DL. Causes and consequences of habitat fragmentation in river networks. Ann N Y Acad Sci. 2015;1355(1): 31–51. doi: 10.1111/nyas.12853 26267672

[3] Perkin JS, Gido KB, Cooper AR, Turner TF, Osborne MJ, Johnson ER, Mayes KB. Fragmentation and dewatering transform Great Plains stream fish communities. Ecol Monogr. 2015;85(1): 73–92.

[4] Schumann DA, Haag JM, Ellensohn PC, Redmond JD, Graeb KNB. Restricted movement of prairie fishes in fragmented riverscapes risks ecosystem structure being ratcheted downstream. Aquat Conserv. 2019;29(2): 235–244.

[5] Januchowski-Hartley SR, McIntyre PB, Diebel M, Doran PJ, Infante DM, Joseph C, Allan JD. Restoring aquatic ecosystem connectivity requires expanding inventories of both dams and road crossings. Front Ecol Environ. 2013;11(4): 211–217.

[6] Januchowski-Hartley SR, Diebel M, Doran PJ, McIntyre PB. Predicting road culvert passability for migratory fishes. Divers Distrib. 2014;20(12): 1414–1424.

[7] Martin EH. Assessing and prioritizing barriers to aquatic connectivity in the Eastern United States. J Am Water Resour Assoc. 2019;55(2): 401–412.

[8] Perkin JS, Acre MR, Graham J, Hoenke K. An integrative conservation planning framework for aquatic landscapes fragmented by road-stream crossings. Landsc Urban Plan. 2020;202: 103860.

[9] SARP. Southeast Aquatic Connectivity Program. Southeast Aquatic Resources Partnership. 2022. Available from: https://southeastaquatics.net/sarps-programs/southeast-aquatic-connectivity-assessment-program-seacap

[10] Briggs AS, Galarowicz TL. Fish passage through culverts in Central Michigan warmwater streams. N Am J Fish Manag. 2013;33(3): 652–664.

[11] Castro-Santos T. Modeling the effect of varying swim speeds on fish passage through velocity barriers. Trans Am Fish Soc. 2006;135(5): 1230–1237.

[12] Ficke AD, Myrick CA, Jud N. The swimming and jumping ability of three small Great Plains fishes: Implications for fishway design. Trans Am Fish Soc. 2011;140(6): 1521–1531.

[13] Kimball ME, Boswell KM, Rozas LP, Berwaldt EK, Richards AR. Swimming abilities of juvenile estuarine fishes: Implications for passage at water control structures. Wetl Ecol Manag. 2018;26(3): 383–390.

[14] Sánchez-González JR, Morcillo F, Ruiz-Legazpi J, Sanz-Ronda FJ. Fish morphology and passage through velocity barriers. Experience with northern straight-mouth nase (Pseudochondrostoma duriense Coelho, 1985) in an open channel flume. Hydrobiologia. 2021;849(6): 1351–1356.

[15] Goerig E, Castro-Santos T. Is motivation important to brook trout passage through culverts? Can J Fish Aquat Sci. 2017;74(6): 885–893.

[16] Norman JR, Hagler MM, Freeman MC, Freeman BJ. Application of a multistate model to estimate culvert effects on movement of small fishes. Trans Am Fish Soc. 2009;138(4): 826–838.

[17] Blanton RE, Cashner MF, Thomas MR, Brandt SL, Floyd MA. Increased habitat fragmentation leads to isolation among and low genetic diversity within populations of the imperiled Kentucky Arrow Darter (Etheostoma sagitta spilotum). Conserv Genet. 2019;20(5): 1009–1022.

[18] Consuegra S, O’Rorke R, Rodriguez-Barreto D, Fernandez S, Jones J, Garcia de Leaniz C. Impacts of large and small barriers on fish assemblage composition assessed using environmental DNA metabarcoding. Sci Total Environ. 2021;790: 148054. doi: 10.1016/j.scitotenv.2021.148054 34111787

[19] Euclide P, Marsden JE. Role of drainage and barriers in the genetic structuring of a tessellated darter population. Conserv Genet. 2018;19(6): 1379–1392.

[20] Coffman JS. Evaluation of a predictive model for upstream fish passage through culverts. M.Sc. Thesis, James Madison University. 2005.

[21] Poplar‐Jeffers IO, Petty JT, Anderson JT, Kite SJ, Strager MP, Fortney RH. Culvert replacement and stream habitat restoration: Implications from Brook Trout management in an Appalachian watershed, U.S.A. Restor Ecol. 2009;17(3): 404–413.

[22] Furniss M, Love M, Firor S, Moynan K, Llanos A, Guntle J, Gubernick R. FishXing (3.0) [Computer software]. 2006.

[23] Kern P, Cramp RL, Gordos MA, Watson JR, Franklin CE. Measuring Ucrit and endurance: Equipment choice influences estimates of fish swimming performance. J Fish Biol. 2018;92(1): 237–247. doi: 10.1111/jfb.13514 29193071

[24] Wolter C, Arlinghaus R. Navigation impacts on freshwater fish assemblages: The ecological relevance of swimming performance. Rev Fish Biol Fish. 2003;13(1): 63–89.

[25] Scott MK, Magoulick DD. Swimming performance of five warmwater stream fish species. Trans Am Fish Soc. 2008;137(1): 209–215.

[26] Nagle BC, Simons AM. Rapid diversification in the North American minnow genus Nocomis. Mol Phylogenet Evol. 2012; 63(3): 639–649. doi: 10.1016/j.ympev.2012.02.013 22415015

[27] Eads CB, Bringolf RB, Greiner RD, Bogan AE, Levine JF. Fish hosts of the Carolina Heelsplitter (Lasmigona decorata), a federally endangered freshwater mussel (Bivalvia: Unionidae). Am Malacol Bull. 2010;28(2): 151–158.

[28] Sterling KA, Warren ML. Description of a new species of cryptic snubnose darter (Percidae: Etheostomatinae) endemic to north-central Mississippi. PeerJ, 2020;8: e9807. doi: 10.7717/peerj.9807 32944422 PMC7469936

[29] Shields FD, Knight SS, Cooper CM. Rehabilitation of aquatic habitats in warmwater streams damaged by channel incision in Mississippi. Hydrobiologia. 1998;382(1): 63–86.

[30] Sterling KA, Warren ML, Henderson LG. Conservation assessment of the Yazoo Darter (Etheostoma raneyi). Southeast Nat. 2013;12(4): 816–842.

[31] Jelks HL, Walsh SJ, Burkhead NM, Contreras-Balderas S, Diaz-Pardo E, Hendrickson DA, et al. Conservation status of imperiled North American freshwater and diadromous fishes. Fisheries. 2008;33(8): 372–407.

[32] Knight SS, Cooper CM. Fishes of Otoucalofa Creek, Mississippi prior to major channel modifications. J Miss Acad Scie. 1987.

[33] Powers SL, Warren ML. Phylogeography of three Snubnose Darters (Percidae: Subgenus Ulocentra) endemic to the Southeastern U.S. Coastal Plain. Copeia. 2009;2009(3): 523–528.

[34] Sterling KA. Conservation genetics and distribution of the Yazoo Darter (Etheostoma Raneyi). M.Sc. Thesis, The University of Mississippi. 2011. Available from: https://egrove.olemiss.edu/etd/272

[35] Sterling KA, Reed DH, Noonan BP, Warren ML. Genetic effects of habitat fragmentation and population isolation on Etheostoma raneyi (Percidae). Conserv Genet. 2012;13(3): 859–872.

[36] Sterling KA, Nielsen SV, Brown AJ, Warren ML, Noonan BP. Cryptic diversity among Yazoo Darters (Percidae: Etheostoma raneyi) in disjunct watersheds of northern Mississippi. PeerJ. 2020;8: e9014. doi: 10.7717/peerj.9014 32411520 PMC7204820

[37] Jenkins RE, Burkhead NM. The Freshwater Fishes of Virginia. Am Fish Soc. 1993.

[38] Rohde FC, Arndt RG, Foltz JW, Quattro JM. Freshwater Fishes of South Carolina. Univ of South Carolina Press. 2009.

[39] Galang MA, Morris LA, Markewitz D, Jackson CR, Carter EA. Prescribed burning effects on the hydrologic behavior of gullies in the South Carolina Piedmont. For Ecol Manage. 2010;259(10): 1959–1970.

[40] Trimble SW. Man-induced soil erosion on the Southern Piedmont, 1700–1970. 2nd ed. Soil Water Conserv Soc. 2008.

[41] USFWS. Carolina Heelsplitter (Lasmigona decorata) [5-Year Review: Summary and Evaluation]. 2019.

[42] Brett JR. The respiratory metabolism and swimming performance of young Sockeye Salmon. Can J Fish Aquat Sci. 1964;21(5): 1183–1226.

[43] SARP. SARP Culvert Assessment Datasheet. Southeast Aquatic Resources Partnership. 2021. Available from: https://southeastaquatics.net/sarps-programs/southeast-aquatic-connectivity-assessment-program-seacap/culvert-assessments/sarp-culvert-assessment-datasheet/view

[44] Diebel MW, Fedora M, Cogswell S, O’Hanley JR. Effects of road crossings on habitat connectivity for stream-resident fish. River Res Appl. 2015;31(10): 1251–1261.

[45] O’Hanley JR, Wright J, Diebel M, Fedora MA, Soucy CL. Restoring stream habitat connectivity: A proposed method for prioritizing the removal of resident fish passage barriers. J Environ Manage. 2013;125: 19–27. doi: 10.1016/j.jenvman.2013.02.055 23632001

[46] O’Hanley JR, Tomberlin D. Optimizing the removal of small fish passage barriers. EnvironModel Assess. 2005;10(2): 85–98.

[47] Mahlum S, Cote D, Wiersma YF, Kehler D, Clarke KD. Evaluating the barrier assessment technique derived from FishXing software and the upstream movement of Brook Trout through road culverts. Trans Am Fish Soc. 2014;143(1): 39–48.

[48] Near TJ, Bossu CM, Bradburd GS, Carlson RL, Harrington RC, Hollingsworth PR Jr, et al. Phylogeny and temporal diversification of darters (Percidae: Etheostomatinae). Syst Biol. 2011;60(5): 565–595. doi: 10.1093/sysbio/syr052 21775340

[49] Bourne CM, Kehler DG, Wiersma YF, Cote D. Barriers to fish passage and barriers to fish passage assessments: The impact of assessment methods and assumptions on barrier identification and quantification of watershed connectivity. Aquat Ecol. 2011;45(3): 389–403.

[50] Rolls RJ. The role of life-history and location of barriers to migration in the spatial distribution and conservation of fish assemblages in a coastal river system. Biol Conserv. 2011;144(1): 339–349.

[51] Bower LM, Peoples BK, Eddy MC, Scott MC. Quantifying flow–ecology relationships across flow regime class and ecoregions in South Carolina. Sci Total Environ. 2022;802: 149721. doi: 10.1016/j.scitotenv.2021.149721 34454154

[52] Poff NL, Allan JD. Functional Organization of stream fish assemblages in relation to hydrological variability. Ecology, 1995;76(2): 606–627.

[53] Poff NL, Ward JV. Implications of streamflow variability and predictability for lotic community structure: A regional analysis of streamflow patterns. Can J Fish Aquat Sci. 1989;46(10): 1805–1818.

[54] Bunt CM, Castro‐Santos T, Haro A. Performance of fish passage structures at upstream barriers to migration. River Res Appl. 2012;28(4): 457–478.

[55] Naughton GP, Caudill CC, Peery CA, Clabough TS, Jepson MA, Bjornn TC, et al. Experimental evaluation of fishway modifications on the passage behaviour of adult Chinook Salmon and Steelhead at Lower Granite Dam, Snake River, USA. River Res Appl. 2007;23(1): 99–111.

[56] Lee CG, Farrell AP, Lotto A, MacNutt MJ, Hinch SG, Healey MC. The effect of temperature on swimming performance and oxygen consumption in adult Sockeye (Oncorhynchus nerka) and Coho (O. kisutch) Salmon stocks. J Exp Biol. 2003;206(18): 3239–3251. doi: 10.1242/jeb.00547 12909705

[57] Peck MA, Buckley LJ, Bengtson DA. Effects of temperature and body size on the swimming speed of larval and juvenile Atlantic Cod (Gadus morhua): Implications for individual-based modelling. Environ Biol Fishes. 2006;75(4): 419–429.

[58] Rodgers EM, Cramp RL, Gordos M, Weier A, Fairfall S, Riches M, et al. Facilitating upstream passage of small-bodied fishes: Linking the thermal dependence of swimming ability to culvert design. Mar Freshw Res. 2014;65(8): 710–719.

