# Supplementary material for: Integrating fish swimming abilities into rapid road crossing barrier assessment: Case studies in the southeastern United States
Source: PLoS One. 2024 Feb 28;19(2):e0298911. doi: 10.1371/journal.pone.0298911 (PMC10901344; doi:10.1371/journal.pone.0298911)
Supplement: S1 Appendix — (DOCX) [file pone.0298911.s001.docx]

**Appendix**

**A.1**

| **Table A1.** Example road crossing characteristics for illustrating the scoring process for the Southeastern Aquatic Resources Partnership Barrier Prioritization Tool. | | |
| --- | --- | --- |
| **Variable** | **Value (score)** | **Weight** |
| Outlet drop | 0 ft. (1) | 0.161 |
| Physical barriers | None (1) | 0.135 |
| Constriction | Moderate (0.5) | 0.090 |
| Inlet grade | At stream grade (1) | 0.088 |
| Water depth | 0.4 ft. (0.4) | 0.082 |
| Water velocity | Significantly faster (0); 43 cm/s | 0.080 |
| Scour pool | Small (0.8) | 0.071 |
| Substrate matches stream | Contrasting (0.75) | 0.070 |
| Substrate coverage | 75% (0.7) | 0.057 |
| Openness | 0.94 (0.998) | 0.052 |
| Height | 6 ft. (0.97) | 0.045 |
| Outlet armoring | None (1) | 0.037 |
| Internal structures | None (1) | 0.032 |

Here, we provide an example scenario of using the SARP protocol paired with species-specific swimming ability to score a road crossing structure with certain characteristics. Consider a round culvert with a diameter of 6 ft. and a length of 30 ft. The culvert is placed within a creek with an average bankfull width of 10 ft., substrate that is largely composed of gravel, average water velocity of 20 cm/s, and average pool depths and widths of 2 and 4 ft., respectively. The remaining measured characteristics and the weights associated with their sub-scores are listed in the above table.

The culvert has been placed flush with the bottom of the stream, so that there is no outlet drop. The equation for the outlet drop sub-score is

s_od_ = 1 – (1.029412x^2^/[x^2^ + 0.26470588]),

where x is the value of the outlet drop. Inputting an outlet drop value of 0 will result in a sub-score of 1. There is no outlet drop that could reduce fish passage.

There are no physical barriers (e.g., sediment or debris) preventing entrance into or passage through the culvert, resulting in a physical barrier sub-score of 1.

The culvert is defined as being moderately constricted, as the structure width is less than the bankfull width but still greater than 50% of the bankfull width. Structures that are moderately constricted receive sub-score values of 0.5.

The inlet of the culvert is also placed flush with the bottom of the stream and is therefore at stream grade. Fish would have no need to jump up into or fall down into the culvert. The inlet grade sub-score then receives a value of 1.

The culvert has a mean water depth of 0.4 ft. and receives a sub-score of that value.

The mean water velocity throughout the culvert is 43 cm/s. This velocity is significantly faster than that throughout the reference reach (20 cm/s). Structures with significantly faster water velocities than the reference reach receive a sub-score of 0. After having measured fish swimming speeds, it is calculated that a proportion of 0.6 of the individuals of the tested species had swimming speeds at least as great as the water velocity flowing through the structure. This proportion is not used in the SARP standard protocol but can be integrated into the protocol as further described below in the final scoring process.

There is a pool at both the inlet and the outlet of the culvert with a depth of 3 ft. and a width of 5ft. Because these scour pools are greater in depth and width (but not at least two times greater) than pools in the reference reach, these are considered small scour pools, and the scour pool sub-score receives a value of 0.8.

The substrate within the culvert is primarily composed of sand, whereas that within the reference reach is primarily composed of gravel. Therefore, the substrate is considered contrasting, and the substrate matches stream sub-score receives a value of 0.75. The substrate forms a continuous, side-to-side coverage of the bottom of the culvert for approximately 75% of the culvert length, which results in a substrate coverage score of 0.7.

Structure openness is defined as the cross-sectional area of the structure divided by its length, so is calculated as follows:

Openness = (π * r^2^)/length,

Openness = (3.14 * 9)/30,

Openness = 0.94.

The equation for the Openness sub-score is

s_o_ = 1(1 – e^-15x(1-0.62)^)^1/(1-0.62)^,

where x is the openness value calculated above. Inputting an openness value of 0.94 into this equation yields an openness sub-score of 0.998.

The equation for the height sub-score is

s_h_ = min[1.1x^2^/(x^2^ + 4.84), 1],

where x is the structure height measured in feet. Inputting a height of 6 ft. into this equation yields a height sub-score of 0.97.

There is no armoring placed outside of the outlet, and there are no structures within the culvert. Therefore, the outlet armoring and internal structure sub-scores both receive values of 1.

With the above sub-scores and their associated weights, we can calculate the overall barrier severity score. With the standard SARP protocol, the barrier severity score is equal to the lesser value between the sum of all weighted sub-scores, and the outlet drop sub-score. With our data, we will be able to add a third criteria so that the overall barrier severity score will be equal to the least value among the sum of all weighted sub-scores, the outlet drop sub-score, and the proportion of tested fish that had swimming speeds at least as high as the measured water velocity. Therefore, the barrier score would be calculated as follows:

Barrier severity score = min[summed weighted scores, outlet drop score, velocity measurement score].

The sum of the weighted scores is calculated as,

Sum weighted scores = (1*0.161) + (1*0.135) + (0.5*0.090) + (1*0.088) + (0.4*0.082) + (0*0.080) + (0.8*0.071) + (0.75*0.07) + (0.7*0.057) + (0.998*0.052) + (0.97*0.045) + (1*0.037) + (1*0.032),

Sum weighed scores = 0.78.

The outlet drop sub-score is 1, and the proportion of tested fish with a swimming speed at least as great as the water velocity through the structure is 0.6. Therefore, the final barrier score is calculated as,

Barrier severity score = min[0.78, 1, 0.60],

Barrier severity score = 0.60.

Without including the proportion of fish that swam at least as fast as the measured water velocity (0.6), the overall barrier score would have been calculated as a higher 0.78. Including that metric yields a different estimate of passability than derived by the standard SARP protocol.
