# Supplementary material for: Integrating fish swimming abilities into rapid road crossing barrier assessment: Case studies in the southeastern United States
Source: PLoS One. 2024 Feb 28;19(2):e0298911. doi: 10.1371/journal.pone.0298911 (PMC10901344; doi:10.1371/journal.pone.0298911)
Supplement: S1 Table — (DOCX) [file pone.0298911.s002.docx]

| **S2 Table.** T-test results for paired comparisons of barrier scores before and after integrating *U_crit_* in each watershed. | | | | | |
| --- | --- | --- | --- | --- | --- |
| **Watershed** | **Difference** | | **Degrees of freedom** | ***t*** | ***p*** |
|  | **mean** | **SE** |  |  |  |
| Stevens Creek | 0.0171 | 0.0118 | 25 | 1.443 | 0.1613 |
| Yocona River | 0.0838 | 0.0358 | 33 | 2.344 | 0.0252 |
