# Supplementary material for: Integrating fish swimming abilities into rapid road crossing barrier assessment: Case studies in the southeastern United States
Source: PLoS One. 2024 Feb 28;19(2):e0298911. doi: 10.1371/journal.pone.0298911 (PMC10901344; doi:10.1371/journal.pone.0298911)
Supplement: S2 Table — (DOCX) [file pone.0298911.s003.docx]

| **S3 Table.** Overall scores for all measured road crossings. Local ID corresponds to identifiers used in the SARP database. Latitude and Longitude are given in decimal degrees. SARP score represents the overall barrier score before integrating species-specific *U_crit_*. *U_crit_* score represents the overall barrier score after integrating species-specific *U_crit_*. | | | | | | |
| --- | --- | --- | --- | --- | --- | --- |
| **Watershed** | **Local ID** | **Latitude** | **Longitude** | **SARP score** | ***U_crit_* score** | |
| **Yocona River** | MATT16 | 34.17230205 | -89.43670251 | 0.81 | 0.81 |  |
|  | MATT4 | 34.15594141 | -89.50206707 | 0.00 | 0.00 |  |
|  | RAN1153 | 34.18373228 | -89.55693229 | 0.64 | 0.64 |  |
|  | RAN204 | 34.12848598 | -89.39376272 | 0.16 | 0.16 |  |
|  | RAN205 | 34.13438483 | -89.40157009 | 0.02 | 0.02 |  |
|  | RAN206 | 34.1507166 | -89.42275289 | 0.41 | 0.41 |  |
|  | RAN207 | 34.15671448 | -89.43071257 | 0.00 | 0.00 |  |
|  | RAN208 | 34.19849964 | -89.475208 | 0.19 | 0.19 |  |
|  | RAN210 | 34.16644234 | -89.44373021 | 0.74 | 0.74 |  |
|  | RAN211 | 34.18040275 | -89.46097116 | 0.80 | 0.80 |  |
|  | RAN213 | 34.13300537 | -89.40214448 | 0.72 | 0.72 |  |
|  | RAN214 | 34.1536458 | -89.43316569 | 0.67 | 0.67 |  |
|  | RAN215 | 34.15701229 | -89.43533905 | 0.94 | 0.94 |  |
|  | RAN220 | 34.23917255 | -89.52984925 | 0.02 | 0.02 |  |
|  | RAN262 | 34.20629721 | -89.63843695 | 0.11 | 0.11 |  |
|  | RAN304 | 34.23582245 | -89.58490338 | 0.05 | 0.05 |  |
|  | RAN304B | 34.23679128 | -89.58575928 | 0.81 | 0.06 |  |
|  | RAN350 | 34.2064612 | -89.63640076 | 0.96 | 0.88 |  |
|  | RAN415 | 34.20923338 | -89.54087013 | 0.22 | 0.00 |  |
|  | RAN415B | 34.20800562 | -89.54207748 | 0.75 | 0.06 |  |
|  | RAN442 | 34.20719606 | -89.46551398 | 0.59 | 0.00 |  |
|  | RAN449 | 34.14001015 | -89.54913141 | 0.13 | 0.13 |  |
|  | RAN456 | 34.22725972 | -89.57247456 | 0.49 | 0.49 |  |
|  | RAN461 | 34.19239776 | -89.62726711 | 0.68 | 0.68 |  |
|  | RAN461B | 34.19180231 | -89.62710829 | 0.48 | 0.00 |  |
|  | RAN489 | 34.2348285 | -89.49097476 | 0.61 | 0.61 |  |
|  | RAN632 | 34.15046388 | -89.44562513 | 0.16 | 0.13 |  |
|  | RAN65 | 34.15678327 | -89.54856411 | 0.01 | 0.01 |  |
|  | RAN652 | 34.13792124 | -89.42245884 | 0.50 | 0.50 |  |
|  | RAN668953 | 34.16250264 | -89.53433602 | 0.00 | 0.00 |  |
|  | RAN67 | 34.16041804 | -89.54046458 | 0.04 | 0.04 |  |
|  | RAN69 | 34.17461772 | -89.49640824 | 0.08 | 0.08 |  |
|  | RAN70 | 34.17350764 | -89.50110348 | 0.08 | 0.08 |  |
|  | RAN808 | 34.16109003 | -89.48625895 | 0.76 | 0.76 |  |
| **Stevens Creek** | CUBHC001 | 34.11242122 | -82.11668226 | 0.56 | 0.56 |  |
|  | CUBHC002 | 34.01355352 | -82.1457248 | 0.63 | 0.63 |  |
|  | CUBHC003 | 33.99818898 | -82.15251172 | 0.87 | 0.87 |  |
|  | CUBHC004 | 33.9554005 | -82.12582049 | 0.02 | 0.02 |  |
|  | CUBHC005 | 33.96868555 | -82.1390586 | 0.96 | 0.96 |  |
|  | CUBHC006 | 33.84374202 | -82.11962763 | 1.00 | 1.00 |  |
|  | CUBHC007 | 33.86387261 | -82.10006796 | 1.00 | 1.00 |  |
|  | CUBHC008 | 33.90166028 | -82.11980765 | 0.55 | 0.55 |  |
|  | CUBHC009 | 33.85730675 | -82.18651143 | 1.00 | 1.00 |  |
|  | CUBHC010 | 33.88106476 | -82.13548245 | 0.03 | 0.03 |  |
|  | CUBHC011 | 33.86028469 | -82.0485487 | 1.00 | 0.78 |  |
|  | CUBHC012 | 33.88863085 | -82.04755339 | 0.19 | 0.19 |  |
|  | CUBHC013 | 33.98304211 | -81.99662477 | 0.72 | 0.72 |  |
|  | CUBHC014 | 33.98954146 | -82.06863197 | 0.72 | 0.72 |  |
|  | CUBHC017 | 33.92438319 | -82.19481999 | 0.74 | 0.74 |  |
|  | CUBHC15 | 34.09037771 | -82.11644936 | 0.79 | 0.79 |  |
|  | CUBHC16 | 33.91619792 | -82.17792053 | 0.59 | 0.59 |  |
|  | CUBHC18 | 34.0046876 | -82.12639485 | 1.00 | 1.00 |  |
|  | CUBHC19 | 34.00103689 | -82.01277238 | 0.72 | 0.72 |  |
|  | CUBHC20 | 34.01509261 | -81.96275321 | 1.00 | 1.00 |  |
|  | CUBHC21 | 33.99307589 | -81.94281894 | 1.00 | 0.78 |  |
|  | CUBHC22 | 33.97204285 | -81.91323556 | 0.86 | 0.86 |  |
|  | CUBHC23 | 33.96796053 | -81.90293624 | 0.50 | 0.50 |  |
|  | CUBHC24 | 33.67354272 | -81.97901061 | 0.59 | 0.59 |  |
|  | CUBHC25 | 33.6701289 | -82.02119377 | 1.00 | 1.00 |  |
|  | CUBHC26 | 33.63874084 | -82.10752115 | 0.84 | 0.84 |  |
